# Assessing the public acceptability of proposed policy interventions to reduce the misuse of antibiotics in Australia: A report on two community juries

**DOI:** 10.1111/hex.12589

**Published:** 2017-06-30

**Authors:** Chris Degeling, Jane Johnson, Jon Iredell, Ky‐Anh Nguyen, Jacqueline M. Norris, John D. Turnidge, Angus Dawson, Stacy M. Carter, Gwendolyn L. Gilbert

**Affiliations:** ^1^ Centre for Values, Ethics and the Law in Medicine School of Public Health University of Sydney Sydney NSW Australia; ^2^ Marie Bashir Institute for Infectious Disease and Biosecurity University of Sydney Sydney NSW Australia; ^3^ Centre for Infectious Diseases and Microbiology Westmead Hospital Westmead NSW Australia; ^4^ Institute of Dental Research Westmead Centre for Oral Health and Westmead Institute for Medical Research Sydney NSW Australia; ^5^ Discipline of Life Sciences Faculty of Dentistry University of Sydney Sydney NSW Australia; ^6^ Faculty of Science Sydney School of Veterinary Science University of Sydney Sydney NSW Australia; ^7^ Departments of Pathology, and Molecular and Cellular Biology University of Adelaide Adelaide SA Australia

**Keywords:** antimicrobial resistance, deliberative methods, health policy, one health, primary health care

## Abstract

**Objective:**

To elicit the views of well‐informed community members on the acceptability of proposed policy interventions designed to improve community use of antibiotics in Australia.

**Design:**

Two community juries held in 2016.

**Setting and participants:**

Western Sydney and Dubbo communities in NSW, Australia. Twenty‐nine participants of diverse social and cultural backgrounds, mixed genders and ages recruited via public advertising: one jury was drawn from a large metropolitan setting; the other from a regional/rural setting.

**Main outcome measure:**

Jury verdict and rationale in response to a prioritization task and structured questions.

**Results:**

Both juries concluded that potential policy interventions to curb antibiotic misuse in the community should be directed towards: (i) ensuring that the public and prescribers were better educated about the dangers of antibiotic resistance; (ii) making community‐based human and animal health‐care practitioners accountable for their prescribing decisions. Patient‐centred approaches such as delayed prescribing were seen as less acceptable than prescriber‐centred approaches; both juries completely rejected any proposal to decrease consumer demand by increasing antibiotic prices.

**Conclusion:**

These informed citizens acknowledged the importance of raising public awareness of the risks, impacts and costs of antibiotic resistance and placed a high priority on increasing social and professional accountability through restrictive measures. Their overarching aim was that policy interventions should be directed towards creating collective actions and broad social support for changing antibiotic use through establishing and explaining the need for mechanisms to control and support better prescribing by practitioners, while not transferring the burdens, costs and risks of interventions to consumers.

## 1 INTRODUCTION

Antimicrobial resistance (AMR) is a significant threat to human health and well‐being.^1^ AMR affects the lives and livelihoods of millions globally by disrupting health, agricultural and ecological systems.^2^ Antibiotic use is a key driver of AMR—the more antibiotics we use, the more likely it is that resistance will develop, be amplified and spread.^3^ Following publication of the O'Neill Report, the costs of failing to address the challenges of AMR are becoming clearer and strategies to address AMR have been elevated on national and global political agendas—including developing a global action plan.^4^ As their effectiveness declines and antibiotics become a limited resource, it is increasingly clear that further escalations in the level of AMR will lead to broad, sustained and adverse impacts on the health and well‐being of individuals and their communities.

Governments, professional groups and industry stakeholders in Australia have long recognized that curbing antibiotic misuse is essential to fostering sustainable health‐care and agricultural systems.^5^ Until recently, measures taken in Australia to counter AMR have focused on changing prescriber and consumer expectations and behaviours. Professional bodies and health regulators have emphasized: public education campaigns for health‐care providers and consumers;^6^ the institution of antibiotic stewardship programmes in hospitals;^7^ and restricting access to key classes of antibiotics.^8^ Recent reports from Australian Government agencies indicate that significant progress has been made in limiting unnecessary or high‐risk antibiotic use in agriculture^9^ and inappropriate antibiotic prescribing in hospital settings.^10^ However, despite concerted efforts to promote rational antimicrobial use, Australia continues to have one of the highest rates of community use of antibiotics in the world. In Australia, antibiotics are almost entirely sourced from community‐based human and animal health‐care providers such as general practitioners (GPs), dentists and veterinarians. While available data indicate that per head, the use of antibiotics for animal health is very low by international standards,^11^ recent reports indicate human antibiotic use in Australia is twice that of comparable countries (eg, the Netherlands and Sweden), with no measurable population health benefit.^10^

In June 2015, the *National Antimicrobial Resistance Strategy 2015‐2019* was jointly launched by the Australian Federal Government Ministers of Health and Agriculture^12^ following extended cross‐sectoral consultation. The first two objectives of this seven‐point plan are to:

1. Increase awareness and understanding of AMR through effective communication, education and training; and
2. Implement effective stewardship programmes across human and animal health care to ensure judicious antimicrobial use.

The focus of early action is attenuating demand for antibiotics, while providing practitioners with support to prescribe appropriately through creation of guidelines and decision tools. While the measures introduced under the auspices of the *National Antimicrobial Resistance Strategy* are not overly restrictive and do not directly impinge on practitioner's clinical autonomy, moves towards implementation have encouraged debate within the Australian health system and consideration of a range of policy interventions and new approaches to managing antibiotic misuse in community settings.

Globally, the problem of AMR is increasingly conceptualized as one of supply and demand. Efforts to increase supply are focused on incentivizing therapeutic innovation.^4^ Efforts to attenuate demand are typically focused on education and reducing uncertainty by developing improved diagnostic methods. Despite the logic and appeal of focusing on prescriber and public education about AMR, efforts so far have not delivered the desired impacts.^13^ Systematic evaluations of education campaigns on rational medicine use show their effects are typically short‐lived.^14^ Because the social norms, incentives and structures that drive misuse of medicines almost always remain unchanged, providers and consumers soon revert to previous behaviours once the intervention ends. The disappointing results of educational interventions have led economists, ethicists and public health practitioners to focus on features of demand‐side economics—such as price elasticity and opportunity costs—by proposing a tax on antibiotic use,^15^, ^16^ especially in sectors that are more price‐sensitive such as animal health and agriculture.^17^

Responding to the need to curb antibiotic overuse and misuse, researchers around the world have been trialing more restrictive measures and clinician‐ and patient‐centred behavioural interventions. Policies aimed at postponing access to antibiotics for “low risk” or otherwise demanding patients include post‐dated or delayed prescribing.^14^, ^18^ Interventions aimed at changing clinical decision making include surveillance and peer‐monitoring of prescribers.^19^, ^20^ At their most restrictive, these involve requiring providers to write a justification on the patient record every time antibiotics are prescribed.^20^ This is being contrasted with the effectiveness of less restrictive measures such as encouraging consumers and practitioners to be mindful of AMR by displaying in waiting rooms a “practitioner's pledge” to only prescribe antibiotics when needed.^21^

Clearly, most of the proposed policy remedies described above are not cost or effort neutral. Some are likely to be burdensome and contentious in that they will intentionally, and sometimes coercively, impose new costs on some for the benefit of others.^22^ Because many of the proposed interventions for dealing with AMR also raise questions of fairness, legitimacy, and the common good, implementing them successfully is likely to require significant levels of public support and understanding.^23^

We report on two community juries convened in 2016 to consider how to make antibiotic use in Australia more sustainable. A community jury (similar to the proprietary method Citizens' Juries) is a group of citizens brought together to receive detailed evidence about and deliberate on a specific issue. Community juries have been used in Australia and elsewhere to consider complex and contentious issues surrounding health resource prioritization and the introduction of new health technologies.^24^ Our aim was not to capture front‐of‐mind opinions, but rather to ascertain what a well‐informed citizenry would accept as legitimate policy interventions to curb antibiotic misuse, and why. Community juries are an established, appropriate method to achieve this.^25^ Community juries are designed to promote participant inclusivity and deliberative participation rather than statistical representation. For this reason, a jury is typically comprised of 12‐15 people so that the quality of participation and deliberation is optimized.^26^, ^27^ Unlike surveys and focus groups, they involve extensive provision of information, constructive, structured dialogue between publics and experts, and adequate time for consideration. The method assumes that people can think rationally and change their views should the evidence warrant it. The process is like a legal proceeding, but the outputs are not legally binding: instead, they provide evidence for policymaking.

We consulted relevant policymakers (representatives of the Office of Health Protection, Australian Commission on Safety and Quality in Health Care, Department of Agriculture) to design the questions juries would consider (Box 1). All agreed the key issue to be explored was how we should limit antibiotic use in the Australian community. Based on this consultation and review of the available policy, peer‐reviewed and grey literatures, seven interventions were chosen for the juries to consider.^14^, ^28^ In the light of work currently being undertaken as part of the *National Antimicrobial Resistance Strategy*,^12^ we sought information on what selected members of the public, from metropolitan and regional/rural settings, consider to be the most fair and legitimate means of antibiotic use in the Australian community, and what other policy measures they thought should also be considered. Our study was approved by HREC:BLINDED.

Box 1 **The questions posed to juries**

1 Antibiotics are often used when they are not needed. If this situation continues, antibiotics will no longer be effective when they are needed to treat a serious infection that could be fatal without effective treatment. The following measures have been proposed to help make sure that antibiotics continue to be effective when we really need them.

1. Educate prescribers and the public on appropriate antibiotic use.
2. Make antibiotics significantly more expensive (this additional cost would sometimes be paid by the consumer and sometimes by the taxpayer).
3. Prohibit antibiotics from being dispensed on the day of prescription (ie, require them to be dispensed 3 or more days later)—this could apply to prescriptions written by GPs, veterinarians and/or dentists.
4. Prohibit or severely restrict community‐based practitioners such as GPs, veterinarians and/or dentists from prescribing antibiotics of last resort.
5. Ban the use of growth promoting antibiotics in food‐producing animals.
6. Ask GPs, veterinarians and/or dentists to hang a signed poster in their consulting room pledging to only prescribe antibiotics when they are needed.
7. Require GPs, veterinarians and/or dentists to write a justification on the patient's record each time they prescribe antibiotics.

Part A: Are there any other measures to promote sustainable antibiotic use that the jury thinks should be added to the list for consideration?

In Part B, you will decide which of these seven measures is the best way to make antibiotic use more sustainable. Before you rank these seven measures, you may want to add more to the list.

Part B: In this task, we are asking the jury to reorganize the list of measures. Put the best measure to make antibiotic use more sustainable at the top, and the worst measure at the bottom. Put all of the measures in order, from best to worst. Please carefully record the reasons for your decisions.

## 2 METHODS

Community juries are a deliberative method, with these general characteristics:

1. A group of citizens is convened for 1‐3 days;
2. They are asked to consider a specific issue;
3. They hear evidence from, and ask question of (often opposed) experts;
4. They are given time for deliberation, and to come to a conclusion, which is documented.

There are two main approaches to community juries. In the first, participants work as a group to draft open sets of recommendations on an issue; in the second, jury members vote on options presented by researchers.^29^ We used a combined approach. Each jury was presented with the same list of seven options, followed by a two‐part question for consideration. In Part A of the question, jurors were asked to nominate three policy interventions worthy of consideration but not listed; in Part B, they were then asked to prioritize all ten interventions relative to each other and give reasons for their decisions (Box 1).

### 2.1 Recruitment and selection

We recruited two Community Juries, of both genders and a range of ages (Table 1). We placed advertisements and stories in mass and social media in the Western Sydney and Central Plains areas of NSW Australia. Western Sydney is a large multicultural urban community of 2 million people; Dubbo is a regional centre of 37 000 people and one of the agricultural hubs of central NSW. Metropolitan and regional/rural sites for the juries were chosen because changing the availability and uses of antibiotics has different implications for individuals and their communities in each setting. Changing how antibiotics can be used in agricultural industries, for example, will require an overhaul of animal production systems in rural communities which will likely also lead to the repricing of many foods in urban settings. At the level of individual experiences patients in regional and remote communities often need to wait longer and travel much further to see a community‐based health professional than people living in a large city, so any additional barrier to accessing antibiotics may place additional burdens on these individuals.

**Table 1:** Characteristics of jury participants

	Jury 1 (n=14^a^)	Jury 2 (n=15)
Age (y)		
<40	4	6
40‐70	7	8
>70	3	1
Range	25‐71	23‐70
Median	49.3	47.7
Gender		
Male	6	6
Female	8	9
Highest educational attainment		
High school	4	3
Trade/diploma	3	6
Bachelor degree	5	4
Postgraduate degree	2	2
Cultural background/ethnicity^b^		
Australian	4	9
Southern/Eastern European	3	
South‐East Asian	1	
North‐East Asian	1	
Southern/Central Asian		
North‐West European	2	6
North African	2	
Socio‐economic status of suburb^c^		
Low	6	6
Middle	5	9
High	3	

a One juror pulled out because of illness during the jury in Sydney.

b Based on Australian Standard Classification of Cultural and Ethnic Groups (ASCEG).

c Based on Socio‐economic Index for Area (SEIFA).

Of 82 responders 32 could not commit or were not available. Six were ineligible because they or a close family member had prescribing authority or had recently worked for a pharmaceutical company. Of the remaining sample we recruited 30 jurors (15 from each area) based on their eligibility, socio‐demographic characteristics and availability. We sought socioeconomic and cultural diversity across juries. The jury in Western Sydney was more socio‐culturally diverse than the jury in Dubbo reflecting the composition of these communities; both juries broadly matched the average educational attainment in the Australian population (Table 1). All jurors received a modest honorarium in recognition of their participation and contribution to jury processes and outcomes.

Each jury commenced with an orientation session, to introduce the process and questions for consideration. Participant's queries or concerns were addressed during this introductory session, at the end of which written consent was sought and received from each juror. Jury Day 1 (Saturday) focused on interrogating the evidence and understanding the ethical, legal and practical issues. Jurors were first shown a documentary (30 minute) on the nature of antibiotic resistance, produced by the Science Unit of the Australian Broadcasting Corporation.^30^ Testimony from a range of relevant experts was pre‐recorded and shown to jurors as video presentations. Experts were selected on the basis of their institutional roles, experience and expertise. Expert testimony and jury process were explicitly designed around providing jurors with balanced and factual information rather than creating oppositions—ie, a case “for” or “against” adopting specific interventions. Through these presentations, jurors were provided with expert reviews of: (i) the current regulatory landscape surrounding antibiotic use in Australia, (ii) the nature and mechanism of each of the seven proposed policy interventions; and (iii) the purpose, context and benefits and harms of antibiotic use in community‐based human, animal and dental health‐care practices (Table 2). As part of their witness briefs, all of the professional experts were also asked to draw on their expertise and the best available evidence to describe the likely impacts and implications of each of the proposed policy interventions for their prescriber group, patients and the broader community. Each presentation ran for ~20 minutes. Pre‐recording ensured the format of the evidence presented was standardized. Each expert's bio‐sketch (including descriptions of their institutional roles and relevant clinical experience) and the video presentations shown to the juries are available online.^31^

**Table 2:** Expert testimony provided to Western Sydney and Dubbo community juries

	Expertise	Expert area	Data provided
1	Infectious disease and clinical microbiology	Current measures and progress towards changing antibiotic use in Australia	(i) Review of the regulatory landscape surrounding antibiotic use, and the success of otherwise of current measures being used to curb antibiotic misuse in Australia (ii) A detailed description of the nature and mechanism of each of the proposed policy interventions
2	Infectious disease physician and clinical microbiology	Human health perspectives on managing AMR	(i) Review of the purpose, context and benefits and harms of antibiotic use in community‐based human health‐care practices (ii) Their expert opinion as to the likely impacts and implications of each of the proposed policy interventions for doctors, patients and the broader community
3	Clinical veterinary medicine and veterinary microbiology	Animal health perspectives on managing AMR	(i) Review of the purpose, context, and benefits and harms of antibiotic use in animal health‐care practices (ii) Their expert opinion as to the likely impacts and implications of each of the proposed policy interventions for veterinarians, animals and the broader community
4	Clinical dentistry and oral biology	Oral health perspectives on managing AMR	(i) Review of the purpose, context and benefits and harms of antibiotic use in dental health‐care practices (ii) Their expert opinion as to the likely impacts and implications of each of the proposed policy interventions for dentists, patients and the broader community
5	Political philosophy and public health ethics	Ethical perspectives on managing AMR	(i) The nature of different distribution systems for limited resources (ii) The ethical implications of each of the proposed policy interventions

Immediately after each video, the expert was available by teleconference call or in person for jurors to question. These question and answer sessions, facilitated by a researcher, allowed jurors to clarify or question the arguments presented.

For the first hour of Jury Day 2 (Sunday), jurors reflected on, discussed and debated the evidence, aided by a researcher acting as facilitator. Facilitation focused on promoting constructive dialogue and fair interaction amongst jurors. Juries then deliberated for an hour without researchers present to reach a verdict. After this hour, the researchers re‐entered the jury room. The verdict, underpinning reasoning and dissenting views, were reported to the research team in a final facilitated feedback session; the research team focused on recording these as accurately as possible, constantly checking with the jurors for clarification. The transcripts of the unfacilitated deliberation indicate that constructive dialogue and fair interaction continued during un‐facilitated periods. Our research and reporting processes for these Community juries were cross‐checked against the CJChecklist protocol.^32^

### 2.2 Data collection and analysis

The two deliberative groups (juries) are the unit of analysis in this study. All jury deliberations (facilitated and un‐facilitated) and expert question and answer sessions were audio‐recorded and then transcribed by an independent service. During the final session, a researcher recorded the verdict and reasons on a flipchart during the course of discussion. Each point was reviewed by the jury to ensure accuracy and altered at their direction. The transcripts of all sessions were subsequently qualitatively analysed by the first two authors to identify key reasons why jurors prioritized, supported and/or rejected the options they considered. Open coding was used to identify the range of arguments and reasons put forward by the jurors in their deliberations. Authors one and two then used framework analysis to systematically map how different arguments and reasons appeared in the two juries, respectively.^33^ The findings were reviewed and discussed by all authors to reach consensus on interpretation.

## 3 RESULTS

To recap, in their deliberations, the two juries were asked to address a two‐part question (Box 1):

1. Part A: to identify 3 other measures to promote sustainable antibiotic use that they thought should be added to the list of seven policy interventions under consideration.
2. Part B: to rank the resulting list of 10 policy interventions, from best to worst, and give reasons for their decisions.

### 3.1 Part A

The juries in Western Sydney and Dubbo nominated similar additional policy interventions to form part of their deliberations. Both groups added that: (i) greater effort should be made to monitor imported foods for the presence of antibiotic residues and antibiotic resistant bacteria^1^ ; and (ii) that greater regulatory oversight should be considered, in the form of new national registers of prescriber and consumer antibiotic use. While the jury in Sydney conceived of these prescriber and consumer surveillance systems as independent entities, and therefore as two separate policy interventions, the jury in Dubbo proposed and strongly emphasized the need for a single combined prescriber and consumer register as a single intervention. For their third and final intervention, the jury in Dubbo added: (iii) measures to decrease people's exposure to the iatrogenic acquisition of antibiotic resistant pathogens through the minimization of unnecessary hospital‐based human health care, for example by avoiding speculative or low value surgical procedures and increasing the amount of home‐based outpatient care; and minimizing inpatient exposure to AMR by increasing the number of single occupancy rooms.

Interventions considered but ultimately not supported by either jury included the following:

1. Banning the use of antibacterial disinfectants in household cleaning products;
2. Banning of sales promotion for antibiotics across human and animal health care;
3. Mandatory warnings on antibiotic packaging; and
4. Introducing a yearly quota for each person of antibiotics sourced from primary care.

### 3.2 Part B

Most jurors did not actively differentiate between different groups of prescribers (GPs, dentists and veterinarians) during their deliberations or in their decision making, even though they were given several opportunities to do so. Rather than ranking each measure during the unfacilitated deliberation session, both juries independently chose to create priority categories (high priority, secondary priority, low priority, etc.) to which they then allocated each of the 10 interventions (Table 2). These were to be applied across society, rather than being targeted to specific professional groups. Both juries justified this approach to prioritization on the basis that multiple, co‐ordinated and interrelated interventions would be needed to effect meaningful and sustained changes in antibiotic use in Australian community settings.

### 3.3 Reasons given for the rankings

Both juries placed a relatively high priority on efforts to educate prescribers and consumers on appropriate antibiotic use, because they believed raising community awareness was an essential foundation for effective implementation of all other interventions. Acknowledging that efforts at communication around the issue of AMR had not previously made a substantive difference to levels of antibiotic consumption in Australia, jurors thought that targeted social and on‐product marketing campaigns could be used, in concert with more traditional forms of public health communication, to make people more mindful of the costs and risks of AMR. Food labelling, for example, could include information about the amounts of antibiotics used in production; antibacterial cleaning product labels could have a warning that they can promote resistance among bacteria in the environment. Jurors also noted that public messaging about antibiotic misuse should include alternative strategies for self‐management of minor illnesses—noting that unless people were equipped to respond to mild diseases appropriately, they would revert to established patterns of consumption (Table 3).

**Table 3:** The final rankings of the proposed interventions

Of highest priority for the community jury in Western Sydney	Of highest priority for the community jury in Dubbo
Educate prescribers and the public on appropriate antibiotic use	Create an integrated national register to monitor prescriber and consumer antibiotic use (proposed by Dubbo jury only)
	Require GPs, veterinarians and/or dentists to write a justification on the patient's record each time they prescribe antibiotics
***Secondary priorities***	
Require GPs, veterinarians and/or dentists to write a justification on the patient's record each time they prescribe antibiotics	Educate prescribers and the public on appropriate antibiotic use
Prohibit or severely restrict community‐based practitioners such as GPs, veterinarians and/or dentists from prescribing antibiotics of last resort	Prohibit or severely restrict community‐based practitioners such as GPs, veterinarians and/or dentists from prescribing antibiotics of last resort
Create a national register to monitor “prescriber” antibiotic use (proposed by Sydney jury only)	
***Tertiary priorities***	
Ban the use of growth promoting antibiotics in food‐producing animals	Minimization of hospital‐based care (proposed by Dubbo jury only)
Monitoring of imported foods for the presence of resistant organisms and antibiotic residues	Ban the use of growth promoting antibiotics in food‐producing animals
***Low priorities***	
Ask GPs, veterinarians and/or dentists to hang a signed poster in their consulting room pledging to only prescribe antibiotics when they are needed	Ask GPs, veterinarians and/or dentists to hang a signed poster in their consulting room pledging to only prescribe antibiotics when they are needed
National register to monitor “consumer” antibiotic use (proposed by Sydney jury only)	Monitoring of imported foods for the presence of resistant organisms and antibiotic residues
Prohibit antibiotics from being dispensed on the day of prescription (ie, require them to be dispensed 3 or more days later)	Prohibit antibiotics from being dispensed on the day of prescription (ie, require them to be dispensed 3 or more days later)
***Should not be considered***	
Make antibiotics significantly more expensive	Make antibiotics significantly more expensive

The introduction of surveillance systems and more robust restrictions to monitor and modify the prescribing behaviours of community‐based practitioners was also strongly supported by both groups. Jurors reasoned that, as trained professionals, human and animal health‐care providers should accept the external auditing of, and take responsibility for, their prescribing decisions. Most jurors did not think these types of measures placed an unwarranted burden on practitioners. In contrast, introducing a “practitioner's pledge” to waiting rooms or delayed prescribing was given a relatively low priority by both juries. Both groups saw the value of reminding everyone of the importance of changing their antibiotic use. Nevertheless, these interventions were not viewed favourably because jurors believed they would be largely unnecessary if the education campaign was effective and the new restrictions on prescribers were properly embedded and accepted by the public. They also thought that unless adopted universally, the “practitioner's pledge” and delayed prescribing would just encourage “doctor shopping” and punish health‐care practitioners who were doing the right thing by their patients and the community.

The continued use of antibiotics for growth promotion in food production was not supported by either jury. Jurors reasoned that banning the non‐therapeutic use of antibiotics in agriculture was worth the extra cost to consumers. Both juries also thought that stricter monitoring of imported foods for the presence of resistant organisms and antibiotic residues, with trade suspensions for compliance failures, were necessary so that local producers were not unfairly disadvantaged. Both groups saw maintaining the capacity to treat infectious disease in animals as important. However, the Western Sydney jury gave a higher priority than the Dubbo jury to monitoring imported foods although, as a group representing a rural community, the latter were more likely to be adversely effected by any asymmetrical changes in these policies. Because the costs of banning growth promotants and increased monitoring of imported foodstuff would almost certainly be passed on to consumers, both groups thought that the public education campaign should explain the need for these measures.

Finally, the juries were unanimous that introducing a tax or price disincentive to curb antibiotic use should not be considered, even if its application was limited to animal health and/or agriculture. They reasoned that increasing the cost of antibiotics would only deepen existing inequities of access to effective health care for those truly in need. In their reasoning jurors again noted that, if appropriate antibiotic prescribing was the norm, then increasing the price to reduce demand could not be considered a viable policy option. Jurors were also concerned that placing the burden on consumers in this manner would not facilitate a genuine culture shift, but rather encourage secondary unauthorized markets in antibiotics.

## 4 DISCUSSION

This study aimed to examine and compare the extent to which groups of informed citizens living in large metropolitan and regional/rural settings would support different policy approaches to curbing the misuse of antibiotics in the Australian community. A range of evidence‐based strategies have been shown to significantly reduce the unnecessary prescribing of antibiotics, and current evidence suggests that community‐based practitioners have a central role in maintaining the efficacy of antibiotics.^14^, ^34^ The current policy climate surrounding the implementation of Australia's new *Antimicrobial Resistance Strategy* means that many of these measures are being trialed,^35^ or at least strongly promoted to Australian human and animal health‐care providers.^36^, ^37^ At the same time, the literature on the knowledge, attitudes and beliefs of the general public about antibiotic resistance is vast and growing rapidly.^38^ To date, however, there has been little to no research as to the public acceptability and perceived legitimacy of policy interventions to decrease unnecessary antibiotic consumption, in Australia or elsewhere.^39^

After 2 days of information and deliberation, both juries placed a high priority on: (i) ensuring that the general public were better educated about AMR and their role in responding to the risks posed; and (ii) introducing coercive and restrictive measures that increase practitioner responsibility for their antibiotic prescribing decisions. The overarching policy aim of both juries was to increase accountability as a means to curb antibiotic misuse. Jurors recommended that health‐care providers be made much more accountable for their prescribing decisions through interventions that place restrictions on their clinical autonomy, systems for surveillance and active auditing of their practices, or both. This suggests that, when well‐informed about the drivers of AMR and risks it poses to Australia's agricultural and health‐care systems, members of public are open to imposing more intrusive or restrictive types of measures than those currently being implemented under the government‐led package of reforms.

When given the opportunity to suggest interventions not included in the original list of seven, both juries recommended and strongly supported creating an antibiotic prescribing surveillance system to improve accountability through monitoring health‐care providers and giving them feedback on their prescribing behaviours. Indeed, both juries favoured introducing a suite of further measures and regulations that would act as a check on prescribers to direct them away from unwarranted antibiotic use. Evidence as to the effectiveness of prescriber‐focused interventions is limited, but growing rapidly,^34^ and the results of studies in Australia are now being published.^40^ Previous reviews have found that auditing systems produce only small changes in prescriber behaviours when used as an isolated intervention.^14^ However, more recent empirical work in the USA and Scandinavia suggests that, when part of a larger suite of interventions, surveillance systems that include audit, feedback and peer comparison did significantly reduce inappropriate antibiotic use.^19^, ^20^ The same multisite study suggested that imposing “justificatory mechanisms” on providers can decrease prescribing rates with only modest changes to practice work flows and practitioner perceptions of clinical autonomy.^20^

Introducing new restrictions on prescribers may meet with resistance from some professional groups in Australia, and different models would be required for different health‐care settings.^41^ However, rather than being an imposition on how health‐care providers practice, jurors tended to construe promoting judicious antibiotic use as a component of a clinician's responsibility to broader society. They argued that if steps such as the active auditing of prescribing and requiring health‐care practitioners to write a justification on the patient record were properly integrated with the public education campaign, this would help to reinforce tighter controls on antibiotic use as part of a “whole of society” solution.

Other less coercive interventions and nudging techniques such as the practitioner's pledge have been found to lower prescribing rates,^21^ but were not given a high priority by either jury. It is important to highlight that jurors were not “against” the waiting room posters, but for them these types of interventions were far less important than combining a strong regulatory environment to control supply with a strong effective public information environment to limit demand.

In general, neither group favoured patient‐based interventions such as price increases and delayed prescribing. In human medicine at least, systematic reviews suggest that delayed prescribing is likely to be one of the most effective interventions, especially for upper respiratory tract infections.^42^ It is also perceived favourably because of its relatively low cost and popularity with “pressured” clinicians who remain uncertain which patients will benefit and concerned about the impacts of complications.^14^ A recent cross‐sectional survey of 730 GPs indicates that Australian practitioners perform well when their knowledge of antibiotic resistance is assessed, but despite this almost 40% of respondents admitted that they prescribe antibiotics to meet patient expectations.^43^ Although there is increasing enthusiasm for delayed prescribing among Australian GPs and health researchers,^40^ the low priority placed on this measure by both juries is consistent with other studies that indicates its broader acceptance will require significant efforts by GPs to build trust and public education to highlight the need for a larger community‐oriented response to “save” antibiotics for those who really need them.^44^, ^45^

Any attempt to decrease consumer demand by imposing a tax or levy to increase antibiotic prices was completely rejected by both groups. However, the jurors' aversion to imposing costs on the public to curb antibiotic overuse was restricted to therapeutic use—for both human and animal patients. Jurors saw any price increase for consumers as being punitive because burdens would be imposed on those who truly needed antibiotics. Both groups were also averse to increasing costs for the therapeutic use of antibiotics for animals because of the risk that this would be a barrier to effective care for some that could result in poorer animal welfare outcomes. That said, both juries saw grounds for further restriction on the non‐therapeutic use of antibiotics in animals and the creation of mechanisms—such as monitoring imports for antibiotic residues and resistant bacteria—to ensure that poor practices elsewhere were not rewarded. Both groups were keen to make Australia a shining example in restricting the non‐therapeutic use of antibiotics in food production use while also ensuring that the cost of antibiotic‐free agriculture was shared across society.

Despite similarities in the intervention rankings, there were some differences in how each of the juries justified their positions. The jury in metropolitan Western Sydney seemed keen to emphasize a non‐punitive form of accountability, which was closely integrated with efforts towards raising public awareness about AMR. In contrast, the jury in regional Dubbo were more comfortable introducing more coercive restrictions and punitive forms of accountability for providers who failed to do the right thing. When viewed in the light of the dilemma between the need for responsible and restrictive use of antibiotics on the one hand, and physicians' obligations to their patients on the other, both sets of jurors were very reluctant to shift the risks and burdens of effective antimicrobial stewardship onto patients. For jurors, it was important that any restrictive measures were implemented judiciously such that none acted as a barrier to timely and effective care.

### 4.1 Limitations of the study

A limitation to this study is that community juries are comprised of small groups of “engaged citizens” whose views may not represent those of the general public. However as two juries in different settings came to similar conclusions, it seems likely our findings are replicable.

## 5 CONCLUSION

With or without a second golden age of drug discovery, the sustainable use of antibiotics is essential; to achieve this a major sociocultural shift will be required that is both constitutive of and supported by new distribution systems that allocate this resource according to individual needs, population benefits, a shared conception of the common good, and values such as equity and fairness.^16^ Australia was an early leader in using regulatory measures to restrict the use of antibiotics of critical importance to human health,^8^ but there has been a reluctance among policymakers to place further restrictions on clinicians and health‐care providers. But the juries were clear: health‐care providers are best placed to curb antibiotic misuse and should accept further restrictions and auditing of their prescribing decisions for the benefit of the broader community.

The overarching aim of both juries, during their deliberations, was that policy interventions for this issue should be directed towards creating collective actions and broad social accountability for antibiotic use. The jury outcomes echo the basic but important observation that if we are to curb inappropriate antibiotic use while also ensuring access when antibiotics are required, then a variety of actions are needed to target different groups in each society.^4^, ^12^ However, the jury verdicts also invite critical reflection by regulators and professional bodies regarding the role of health‐care providers in facilitating or mitigating antibiotic overuse and misuse in community settings. Our results show an informed public may embrace attempts to impose stronger controls on prescribers to curb antibiotic misuse in the community, but this must be integrated into a well‐organized and targeted set of policy interventions.

## CONFLICT OF INTERESTS

None to declare.

## Note

1 While foods imported to Australia are monitored for antibiotic residues, they are not currently monitored for antibiotic resistance.
